# Supplementary material for: Reconstructing meaning from bits of information
Source: Nat Commun. 2019 Feb 25;10:927. doi: 10.1038/s41467-019-08848-0 (PMC6389990; doi:10.1038/s41467-019-08848-0)
Supplement: Supplementary file 1 — Supplementary Information [file 41467_2019_8848_MOESM1_ESM.pdf]

Supplementary information

Reconstructing meaning from bits of information

Kivisaari et al.

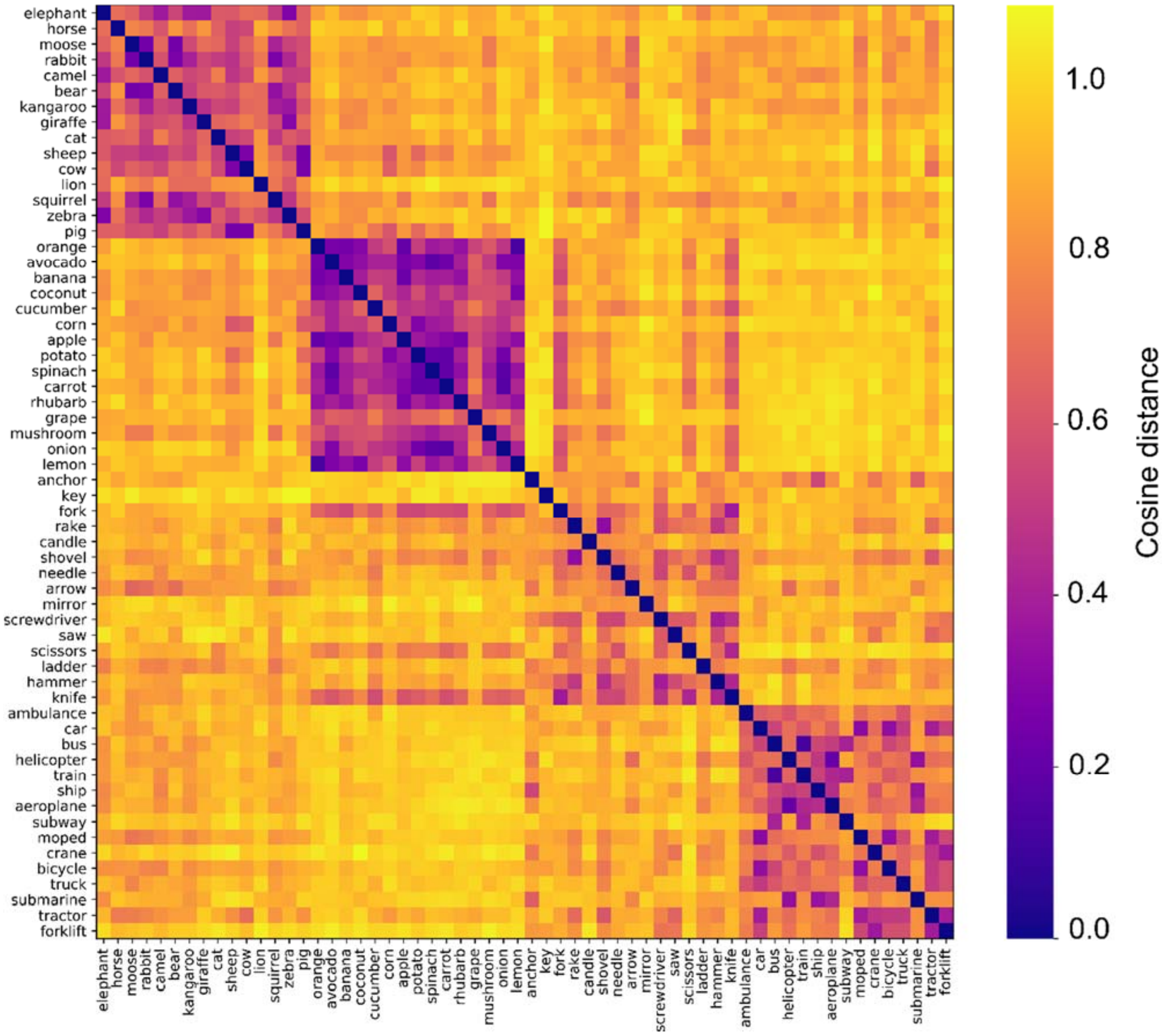

Supplementary Figure 1. The cosine distance between the semantic coordinates of each target item pair based on word2vec.

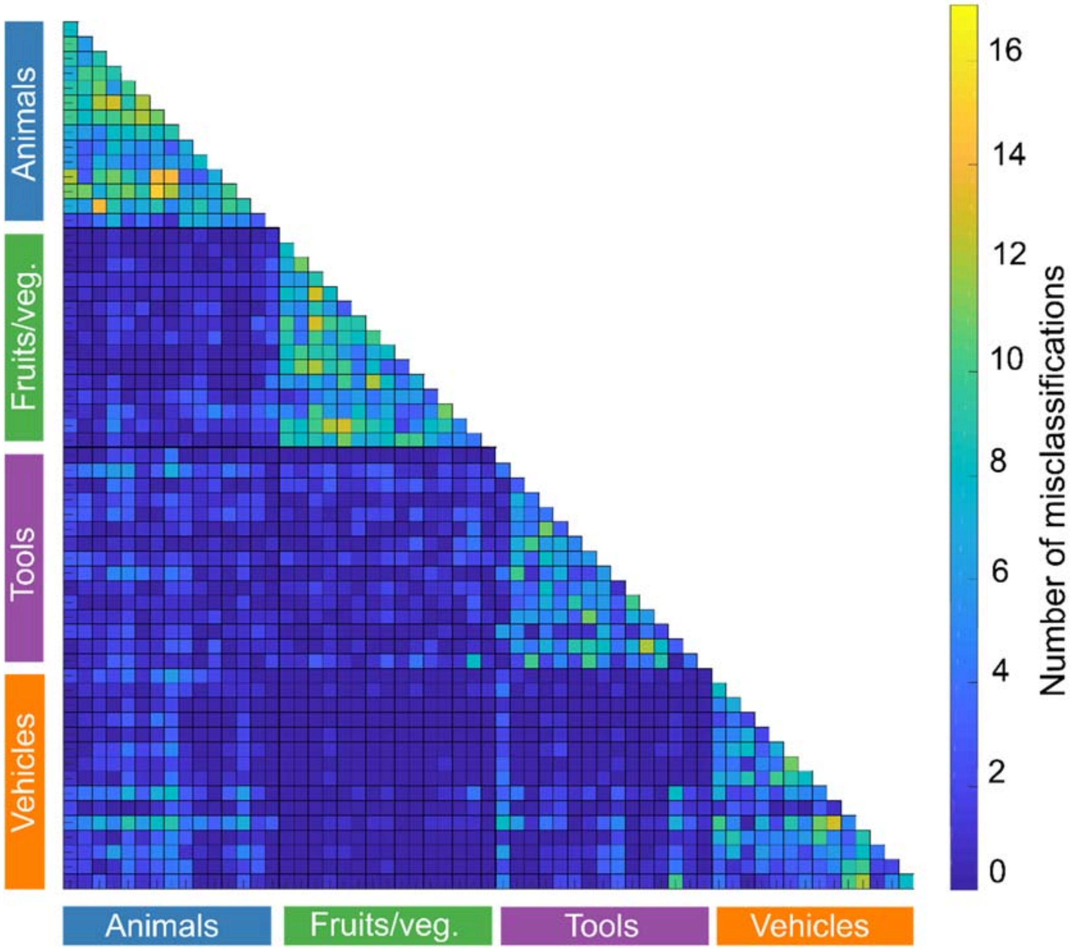

Supplementary Figure 2. The confusion matrix is based on the leave-two-out classification on averaged data. The color scale represents the number of misclassifications in decoding for each target object pair

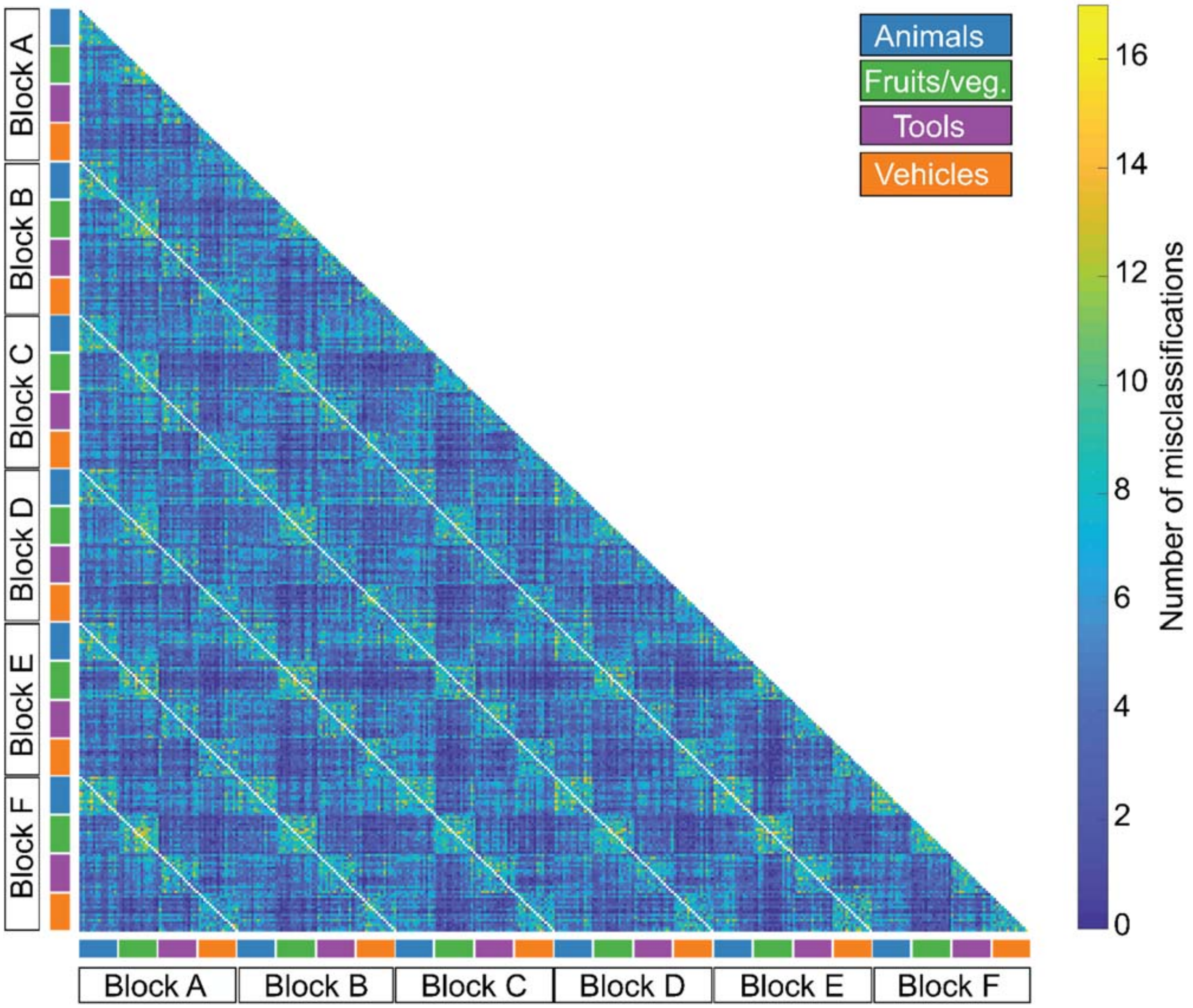

Supplementary Figure 3. The single-trial confusion matrix based on leave-two out classifications. The color-scale represents the number of misclassifications in decoding for each trial pair.

Supplementary Table 1. Machine learning results using averaged data.

participant	overall accuracy	between-category	within-category	naming accuracy
1	91.9	98.6	70.2	96.4
2	93.3	99.3	74.0	98.0
3	92.4	99.3	70.0	91.9
4	82.7	93.0	49.8†	92.5
5	82.1	91.6	51.9†	97.4
6	78.5	85.0	57.6†	91.9
7	84.9	91.6	63.1	90.1
8	88.2	94.7	67.6	94.7
9	89.4	95.8	68.8	94.3
10	89.9	96.1	70.0	88.0
11	87.1	94.7	62.4	87.3
12	87.6	93.7	68.1	91.5
13	85.6	94.7	56.4†	93.8
14	91.9	98.8	69.5	95.2
15	76.1	81.1	60.0†	91.0
16	91.0	99.1	64.8	96.0
17	89.3	94.8	71.4	95.5

†Nonsignificant accuracy. For all other accuracies, $p < 0.05$.

This table shows the machine learning decoding accuracy and naming accuracy by participant. The “within-category” column contains comparisons within a single semantic category (e.g., animal vs. animal or tool vs. tool) whereas the “between-category” column contains comparisons across categories (e.g., animal vs. tool). The “naming accuracy” column indicates the behavioral naming accuracy in the guessing game task in the scanner.

Supplementary Table 2. Table of key resources used in the current study.

Resource	Source	Identifier
Behavioral data	Imaging Language Group, Aalto University	https://aaltoimaginglanguage.github.io/guess/
fMRI data	Imaging Language Group, Aalto University	Available upon request for researchers who meet the criteria for access to confidential data.
MATLAB 2014a	The MathWorks Inc.	https://uk.mathworks.com
Python 3.0	Python Software foundation	www.python.org
scikit-learn module	Pedregosa et al., 2011	http://scikit-learn.org/stable/
Zero-shot decoding code	Imaging Language Group, Aalto University	https://aaltoimaginglanguage.github.io/guess/
RSA toolbox	MRC Cognition and Brain Sciences Unit, University of Cambridge	https://www.mrc-cbu.cam.ac.uk/methods-and-resources/toolboxes
Presentation Software	Neurobehavioral systems	https://www.neurobs.com
SPM8	Wellcome Trust Centre for Neuroimaging, University College London	https://www.fil.ion.ucl.ac.uk/spm/
SnPM13	University of Warwick	http://go.warwick.ac.uk/tenichols/snpm
AAL Atlas	Neurodegenerative Diseases Institute, Université de Bordeaux	http://www.gin.cnrs.fr/en/tools/aal-aal2/
word2vec	Mikolov et al.	https://code.google.com/archive/p/word2vec
Word vector data set	Turku BioNLP Group, University of Turku	http://bionlp-www.utu.fi/fin-vector-space-models
CSLB norm data	Centre for Speech, Language and the Brain	www.csl.psychol.cam.ac.uk/propertynorms
Stimuli	Imaging Language Group, Aalto University	https://aaltoimaginglanguage.github.io/guess/
